# Association analyses of nutritional markers with Parkinson’s disease and Alzheimer’s disease

**DOI:** 10.1016/j.heliyon.2024.e40191

**Published:** 2024-11-06

**Authors:** Dong-Juan Xu, Yi-Lei Shen, Meng-Meng Hu, Ling-Ling Li, Yuan Fang, Ju-Ping He, Lu-Lu Ma, Shan-Shan Xu, Jian-Yong Wang

**Affiliations:** a Department of Neurology, Dongyang Affiliated Hospital of Wenzhou Medical University, Dongyang, Zhejiang, China; b Department of Neurology, Institute of Geriatric Neurology, The Second Affiliated Hospital and Yuying Children’s Hospital, Wenzhou Medical University, Wenzhou, Zhejiang, China

**Keywords:** Parkinson’s disease, Alzheimer’s disease, Homocysteine, Lipid, Apolipoprotein

## Abstract

**Introduction:**

Parkinson’s disease (PD) and Alzheimer’s disease (AD) are common neurodegenerative diseases with multifaceted etiology. Nutritional and metabolic abnormalities are frequently implicated in PD and AD. In this observational study, we analyzed a series of nutritional markers, and aimed to understand their association with AD and PD risk.

**Methods:**

A total of 424 PD patients, 314 AD patients, and 388 healthy controls were included. Nutritional markers including Hemoglobin A1c, vitamin B12, folate, apolipoprotein B (ApoB), apolipoprotein A1 (ApoA1), low-density lipoprotein (LDL), high-density lipoprotein, triglyceride, total cholesterol (TC), uric acid and homocysteine (HCY) were measured. Significance for odds ratios examining was *P* < 0.0045 after bonferroni correction.

**Results:**

Multifactor risk analysis showed that ApoB, LDL, and TC reduce PD risk, while HCY increase PD risk. ApoA1 and HCY are protective and risk factors for AD, respectively.

**Conclusion:**

The cross-sectional study demonstrates that HCY and lipid metabolism markers are associated with PD and AD risks. Our findings support the involvement of one-carbon metabolism and lipid metabolism disturbance in PD and AD.

## Highlights

• Homocysteine and lipid metabolism markers are associated with Parkinson’s disease (PD) and Alzheimer’s disease (AD).
• Apolipoprotein B, low-density lipoprotein, and total cholesterol reduce PD risk, while homocysteine increase PD risk.
• Apolipoprotein A1 is a protective factor for AD, while homocysteine is a risk factor for AD.

## 1 Introduction

Parkinson’s disease (PD) and Alzheimer’s disease (AD) are the 2 most common neurodegenerative diseases worldwide, and their prevalence rises rapidly with age [1]. They are significantly different in clinical manifestations and pathological characteristics. PD is characterized by bradykinesia, tremor, rigidity, and postural instability, mainly resulting from dramatic loss of dopaminergic neurons within the substantia nigra [2]. While AD is characterized by cognitive impairment which is due to the accumulation of abnormally folded amyloid β (Aβ) and tau proteins in patients’ brains [3].

The mechanisms of PD and AD remain unknown. Both of them are believed to be caused by genetic, epigenetic and environmental factors [[4], [5], [6]]. Emerging evidences have shown that nutritional factors are associated with PD and AD, and they can serve as potential targets for intervention in the two diseases. For example, lipid metabolism [7,8], one-carbon metabolism [9], and glucose metabolism [10,11] are considered to be associated with the risk of PD and AD. Furthermore, vitamin B12 supplementation may reduce the risk of PD [12], and B vitamin supplementation may slow the cognitive decline of dementia patients [13].

It is worth noting that nutritional status varies greatly among populations with different demographic characteristics, social status and dietary habit [14]. Nutritional markers are not independent of each other, and metabolic syndromes is often accompanied by changes in different nutrients [15]. Carbohydrate and lipid metabolism influence each other, sharing similar mechanisms and pathways [16]. Many previous studies have explored the relationship between nutritional markers and the risk of PD and AD, but they did not exclude metabolic diseases such as diabetes mellitus and hyperlipidemia when they recruited participants [8,9,11].

In this study, we evaluated a series of nutritional markers in a cohort of 1126 subjects who had no comorbid metabolic disease, and aimed to investigate if any of them were associated with the risk of AD and PD.

## 2 Methods

### 2.1 Patients

A total of 1126 subjects of Han Chinese ethnicity were enrolled in this study, including 388 healthy controls, 424 PD patients and 314 AD patients. All of them were recruited from Department of Neurology, Dongyang Affiliated Hospital of Wenzhou Medical University from March 2013 to July 2022. PD patients were diagnosed according to the UK Parkinson’s disease Society Brain Bank Criteria [17]. AD patients were diagnosed according to the National Institute on Aging/Alzheimer's Association workgroups on the diagnostic guidelines for AD [18]. All of the PD and PD patients were free of metabolic diseases. Healthy controls were volunteers free of neurodegenerative and metabolic diseases. Clinical information including age, gender, disease duration and body mass index (BMI) were assessed by face-to-face interview and physical examinations. Hemoglobin A1c (HbA1c) was detected by ion exchange-high-performance liquid chromatography analysis. The serum levels of vitamin B12, folate, apolipoprotein B (ApoB), apolipoprotein A1 (ApoA1), low-density lipoprotein (LDL), high-density lipoprotein (HDL), triglyceride (TG), total cholesterol (TC), uric acid (UA) and homocysteine (HCY) were measured by electrochemiluminescence immunoassay. All the samples were analyzed immediately after collection.

### 2.2 Data analysis

All the data were analyzed by using IBM SPSS Statistics 19.0 for windows. Kolmogorov-Smirnov test was used for normality test. Differences in age, vitamin B12, HbA1c, ApoB, ApoA1, HDL, TG, UA and HCY were analyzed by Mann-Whitney test. Difference in gender was assessed by Chi square test. Differences in BMI, folate, LDL and TC were analyzed by unpaired two-tailed *t*-test. Odds ratios examining were performed by binary logistic regression model with age, sex and BMI adjustments. Significance for odds ratios examining was *P* < 0.0045 after bonferroni correction. A two-tailed *P* < 0.05 was considered statistically significant for the remaining comparisons.

## 3 Results

### 3.1 Demographic and clinical summary

A total of 424 PD patients, 314 AD patients and 388 healthy controls were included in our study. As showed in Table 1, controls included 215 females and 173 males, PD patients included 206 females and 218 males, and AD patients included 164 females and 150 males. The median age of controls, PD patients and AD patients was 64 (interquartile range, 59–70), 71 (interquartile range, 64–78), and 80 (interquartile range, 72–85) years old, respectively. The median disease duration of PD patients and AD patients was 3 (interquartile range, 1–8) and 2 (interquartile range, 1–4) years, respectively. The BMI of PD patients and AD patients was significantly different from controls (*P* = 0.011 & *P* = 0.038, respectively). The PD and control groups were comparable in levels of vitamin B12, folate, HDL and UA (*P* > 0.05), but not in HbA1c, ApoB, ApoA1, LDL, TG, TC and HCY (*P* < 0.05). The AD and control groups were comparable in levels of vitamin B12 (*P* > 0.05), but not in folate, HbA1c, ApoB, ApoA1, LDL, HDL, TG, TC, UA and HCY (*P* < 0.05).

**Table 1:** Characteristics of patients and controls.

	Control	PD	AD	*P*^d^	*P*^e^
Subject, n	388	424	314	–	–
Age, years (IR)	64 (59–70)	71 (64–78)	80 (72–85)	<0.001^a^	<0.001^a^
Gender, F/M	215/173	206/218	164/150	<0.052^b^	<0.400^b^
Disease duration, years (IR)	–	3 (1–8)	2 (1–4)	–	–
BMI, mean ± SD	22.0 ± 3.0	22.6 ± 3.4	21.2 ± 3.1	0.011^c^	0.038^c^
Vitamin B12, pmol/L (IR)	276.9 (200.2–380.3)	275.9 (189.9–382.5)	252.1 (160.9–353.8)	0.713^a^	0.089^a^
Folate, nmol/L, mean ± SD	21.4 ± 10.3	19.6 ± 9.7	15.9 ± 10.9	0.114^c^	<0.001^c^
HbA1c, % (IR)	5.5 (5.3–5.7)	5.6 (5.3–5.8)	5.6 (5.3–5.9)	0.006^a^	0.001^a^
ApoB, g/L (IR)	0.89 (0.74–1.03)	0.76 (0.60–0.91)	0.77 (0.62–0.95)	<0.001^a^	<0.001^a^
ApoA1, g/L (IR)	1.19 (1.02–1.39)	1.10 (0.91–1.29)	0.95 (0.77–1.12)	<0.001^a^	<0.001^a^
LDL, mmol/L, mean ± SD	2.67 ± 0.77	2.35 ± 0.80	2.32 ± 0.83	<0.001^c^	<0.001^c^
HDL, mmol/L (IR)	1.16 (0.96–1.39)	1.13 (0.95–1.36)	1.03 (0.85–1.22)	0.233^a^	<0.001^a^
TG, mmol/L (IR)	1.29 (0.92–1.78)	1.06 (0.79–1.52)	1.07 (0.84–1.46)	<0.001^a^	<0.001^a^
TC, mmol/L, mean ± SD	4.62 ± 0.91	4.16 ± 0.98	4.05 ± 0.99	<0.001^c^	<0.001^c^
UA, μmol/L (IR)	255.5 (215.0–307.3)	265.5 (215.0–325.8)	271.0 (217.0–353.0)	0.341^a^	<0.047^a^
HCY, μmol/L (IR)	13.1 (10.4–16.0)	15.4 (11.7–21.6)	17.5 (12.2–23.9)	<0.001^a^	<0.001^a^

AD, Alzheimer disease; ApoA1, Apolipoprotein A1; ApoB, Apolipoprotein B; BMI, Body mass index; F, Female; HbA1c, Hemoglobin A1c; HCY, Homocysteine; HDL, High-density lipoprotein; IR, Interquartile range; LDL, Low-density lipoprotein; M, Male; PD, Parkinson’s disease; SD, Standard deviation; TC, Total cholesterol; TG, Triglyceride; UA, Uric acid.

a Analyzed by Mann-Whitney test.

b Analyzed by Chi square test.

c Analyzed by unpaired two-tailed *t*-test.

d Compared between PD and Controls groups.

e Compared between AD and Controls groups.

### 3.2 Odds ratios of the nutritional markers with incident PD and AD

Further analyses were performed with age, sex and BMI adjustments and bonferroni correction. The results showed that ApoB (*P* < 0.001, OR 0.177, 95 % CI 0.080–0.394), LDL (*P* = 0.002, OR 0.699, 95 % CI 0.555–0.881), and TC (*P* < 0.001, OR 0.694, 95 % CI 0.569–0.847) reduced PD risk, and HCY (*P* < 0.001, OR 1.084, 95 % CI 1.049–1.120) conferred an aggravated risk of developing PD (Table 2). While ApoA1 (*P* = 0.002, OR 0.223, 95 % CI 0.086–0.574) reduced AD risk, and HCY (*P* = 0.002, OR 1.065, 95 % CI 1.024–1.109) increased AD risk (Table 3).

**Table 2:** Odds ratios examining the association of nutritional markers with incident Parkinson’s disease.^a^.

Markers	B	*P*	OR	95 % CI	
				Lower	Upper
Vitamin B12	−0.001	0.670	1.000	0.999	1.001
Folate	0.020	0.189	1.020	0.990	1.050
HbA1c	0.553	0.010	1.738	1.143	2.642
ApoB	−1.729	<0.001^b^	0.177	0.080	0.394
ApoA1	−0.285	0.350	0.752	0.413	1.367
LDL	−0.358	0.002^b^	0.699	0.555	0.881
HDL	0.523	0.085	1.687	0.930	3.062
TG	−0.274	0.017	0.760	0.607	0.952
TC	−0.365	<0.001^b^	0.694	0.569	0.847
UA	−0.001	0.422	0.999	0.997	1.001
HCY	0.080	<0.001^b^	1.084	1.049	1.120

ApoA1, Apolipoprotein A1; ApoB, Apolipoprotein B; BMI, Body mass index; CI, Confidence interval; HbA1c, Hemoglobin A1c; HCY, Homocysteine; HDL, High-density lipoprotein; LDL, Low-density lipoprotein; OR, Odds ratio; TC, Total cholesterol; TG, Triglyceride; UA, Uric acid.

a Adjusted with age, sex and BMI.

b *P* < 0.0045.

**Table 3:** Odds ratios examining the association of nutritional markers with incident Alzheimer disease.^a^.

Markers	B	*P*	OR	95 % CI	
				Lower	Upper
Vitamin B12	−0.001	0.543	1.000	0.998	1.001
Folate	0.010	0.646	1.010	0.969	1.053
HbA1c	0.950	0.005	2.586	1.332	5.020
ApoB	−0.122	0.823	0.886	0.305	2.571
ApoA1	−1.503	0.002^b^	0.223	0.086	0.574
LDL	−0.026	0.877	0.974	0.702	1.352
HDL	−1.020	0.028	0.361	0.146	0.894
TG	0.258	0.049	1.294	1.001	1.674
TC	−0.115	0.436	0.891	0.668	1.190
UA	0.003	0.038	1.003	1.000	1.006
HCY	0.063	0.002^b^	1.065	1.024	1.109

ApoA1, Apolipoprotein A1; ApoB, Apolipoprotein B; BMI, Body mass index; CI, Confidence interval; HbA1c, Hemoglobin A1c; HCY, Homocysteine; HDL, High-density lipoprotein; LDL, Low-density lipoprotein; OR, Odds ratio; TC, Total cholesterol; TG, Triglyceride; UA, Uric acid.

a Adjusted with age, sex and BMI.

b *P* < 0.0045.

## 4 Discussion

PD and AD are common neurodegenerative diseases with multiple factors involved in their pathogenesis. Among them, the roles of nutritional and metabolic factors are especially important, because they can be improved by dietary modifications. In this case control study, we evaluated and compared a series of nutritional markers in a cohort of healthy controls, PD and AD patients without metabolic disease. Our results showed that ApoB, LDL, and TC reduce PD risk, while HCY is a risk factor for PD. ApoA1 and HCY are protective and risk factors for AD, respectively.

HCY is a thio-containing amino acid derived from methionine. Studies have shown that elevated HCY can lead to damage to neurons and blood vessels [19]. Epidemiologic studies have also found that increased HCY is associated with systemic vascular diseases and neurodegenerative diseases [20]. It has been widely accepted that high serum HCY level increase the risk of PD and AD [[21], [22], [23], [24], [25], [26]]. Considering the fact that HCY metabolism and serum HCY levels are influenced by factors such as nutrition, drugs, and genetics [27], we excluded those subjects with comorbid metabolic diseases during recruitment. Our results still support that HCY is an independent risk factor for PD and AD.

The metabolism of HCY depends on B vitamins, including vitamin B12, vitamin B6, and folate. Therefore, some studies explored the effect of intervening HCY on the pathogenesis of AD and PD. A hospital-based case-control study showed that low intake of vitamin B6, but not folate, vitamin B12 or riboflavin, was associated with PD risk [28]. Another cohort study also demonstrated that dietary supplementation of vitamin B6, rather than folate and vitamin B12, can reduce the risk of PD [29]. Interestingly, a recent study showed that vitamin B12 supplementation may have a possible protective effect on the development of PD, but higher intake of folate or vitamin B6 could not reduce PD risk [12]. Of course, there are also studies suggesting that supplementing these three B vitamins cannot reduce the risk of PD [12]. We believe that inconsistency of these conclusions is resulted from the metabolic diversity of HCY and the complexity of PD pathogenesis.

Slowing the cognitive decline or reducing the risk of AD by intervening HCY has also received great attention [30,31]. The conclusion seems to be controversial. A previous study showed that plasma HCY was negatively correlated with the dietary intake of B vitamins, and the total B vitamins intake is associated with cognitive function of AD patients [32]. However, there are also studies that did not support that supplementation of folate, vitamin B6 and vitamin B12 can delay the decline of cognitive function in AD patients [33,34]. More researches are needed to draw the conclusion.

Highlighted should be that we found the associations between biomarkers of lipids and the risks of PD and AD. There is no doubt that LDL and TC are critically risk factors for cardiovascular and cerebrovascular diseases, but they have different effects on neurodegenerative diseases. ApoA1 is the major component of HDL particles, and ApoB is the main protein components of non-HDL particles. They are always applied to improve the predictive value of cardiovascular disease. Our results demonstrated that ApoB, LDL and TC reduce PD risk and ApoA1 is a protective factor for AD. Consistent with our results, a meta-analysis suggested that elevated TG, LDL, and TC may be protective factors for PD risk [7]. Another cohort study also demonstrated that higher levels of TC, LDL, TG and ApoB are associated with a lower future risk of PD [35]. Our findings reinforce that moderate levels of hyperlipidemia may protect dopaminergic neurons.

Similarly, quite a few studies have explored the relationship between biomarkers of lipid metabolism and AD risk [8,36]. A previous retrospective longitudinal study with 156 participants showed that higher ApoA1 was associated with faster cognitive decline in AD patients [36], which is inconsistent with our results. Interestingly, the same study revealed that higher cerebrospinal fluid ApoA1 level was related to slower cognitive decline in the same cohort [36]. An animal study also demonstrated that increasing the serum ApoA1 level in mice may alleviate the pathological features of AD [37]. More researches are needed to clarify the relationship between ApoA1 and the risk of AD.

In the current study, we also explored the relationship between UA and HbA1c and the risk of PD and AD, and the results were negative. Both of them have been previously reported to be associated with PD and AD [10,11,38,39]. The inconsistency may be due to our exclusion of participants with comorbid metabolic diseases during recruitment.

Meanwhile, we have to acknowledge that this study lacks longitudinal observation. The presence of dynamic changes of nutritional markers in PD and AD would enhance the reliability of the results.

In summary, our study demonstrates that HCY is a risk factor for PD and AD, and that ApoB, LDL and TC reduce PD risk and ApoA1 is a protective factor for AD. Our findings support the involvement of one-carbon metabolism and lipid metabolism disturbance in PD and AD.

## CRediT authorship contribution statement

**Dong-Juan Xu:** Writing – review & editing, Supervision, Project administration, Conceptualization. **Yi-Lei Shen:** Writing – review & editing, Methodology, Investigation. **Meng-Meng Hu:** Methodology, Investigation. **Ling-Ling Li:** Methodology, Investigation. **Yuan Fang:** Methodology, Investigation. **Ju-Ping He:** Writing – review & editing, Methodology, Investigation. **Lu-Lu Ma:** Software. **Shan-Shan Xu:** Methodology. **Jian-Yong Wang:** Writing – original draft, Supervision, Funding acquisition.

## Ethics statement

This study was approved by the ethics committee of the Dongyang Affiliated Hospital of Wenzhou Medical University, with ethics approval reference 2023-YX-232. All participants signed written informed consents.

## Data availability statement

The study data are included in the article, for further information please contact the corresponding author.

## Funding

The study was supported in part by funding from Zhejiang Provincial Medical Technology Program (2023RC215).

## Declaration of competing interest

The authors declare that they have no known competing financial interests or personal relationships that could have appeared to influence the work reported in this paper.
